# Presence of SARS-CoV-2 Nucleoprotein in Cardiac Tissues of Donors with Negative COVID-19 Molecular Tests

**DOI:** 10.3390/diagnostics11040731

**Published:** 2021-04-20

**Authors:** Gianluca Lorenzo Perrucci, Elena Sommariva, Veronica Ricci, Paola Songia, Yuri D’Alessandra, Paolo Poggio, Giulio Pompilio, Gianluca Polvani, Anna Guarino

**Affiliations:** 1 Unità di Biologia Vascolare e Medicina Rigenerativa, Centro Cardiologico Monzino IRCCS, 20138 Milano, Italy; elena.sommariva@ccfm.it (E.S.); giulio.pompilio@ccfm.it (G.P.); 2 Unità di Proteomica Cardiovascolare, Centro Cardiologico Monzino IRCCS, 20138 Milano, Italy; veronica.ricci@ccfm.it (V.R.); yuri.dalessandra@ccfm.it (Y.D.); 3 Dipartimento di Medicina Clinica e Chirurgia, Università deli Studi Federico II, 80138 Napoli, Italy; 4 Unità per lo Studio delle Patologie Aortiche, Valvolari e Coronariche, Centro Cardiologico Monzino IRCCS, 20138 Milano, Italy; paola.songia@ccfm.it (P.S.); paolo.poggio@ccfm.it (P.P.); 5 Unità di Chirurgia Cardiovascolare, Centro Cardiologico Monzino IRCCS, 20138 Milano, Italy; gianluca.polvani@ccfm.it; 6 Dipartimento di Scienze Biomediche, Chirurgiche ed Odontoiatriche, Università degli Studi, 20122 Milano, Italy; 7 Banca Tessuti Cardiovascolari, Regione Lombardia, Centro Cardiologico Monzino IRCCS, 20138 Milano, Italy; anna.guarino@ccfm.it

**Keywords:** SARS-CoV-2 nucleoprotein, tissue donor transplantation, pulmonary vein wall, cardiac tissue, diagnosis

## Abstract

The 2019 Coronavirus disease (COVID-19) outbreak had detrimental effects on essential medical services such as organ and tissue donation. Lombardy, one of the most active Italian regions in organ/tissue procurement, has been strongly affected by the COVID-19 pandemic. To date, data concerning the risk of SARS-CoV-2 transmission after tissue transplantation are controversial. Here, we aimed to evaluate the presence/absence of SARS-CoV-2 in different cardiac tissues eligible for transplantation obtained from Lombard donors. We used cardiovascular tissues from eight donors potentially suitable for pulmonary valve transplantation. All donor subjects involved in the study returned negative results for the SARS-CoV-2 RNA molecular tests (quantitative real-time reverse-transcription PCR, qRT-PCR, and chip-based digital PCR) in nasopharyngeal swabs (NPS) or bronchoalveolar lavage (BAL). None of the eight donors included in this study revealed the presence of the SARS-CoV-2 viral genome. However, evaluation of the protein content of pulmonary vein wall (PVW) tissue revealed variable levels of SARS-CoV-2 nucleoprotein signal in all donors. Our study demonstrated for the first time, to the best of our knowledge, that viral nucleoprotein but not viral RNA was present in the examined tissue bank specimens, suggesting the need for caution and in-depth investigations on implantable tissue specimens collected during the COVID-19 pandemic period.

## 1. Introduction

The Coronavirus 2019 (COVID-19) outbreak has not only caused global social disruptions and direct worldwide effects on hospitals, but has also indirectly impacted several essential areas of the healthcare system, such as organ and tissue donation for transplant [1]. Lombardy, one of the most active Italian regions in organ/tissue procurement, has been the most affected by the COVID-19 pandemic [2]. On February 24 th, the Italian Transplant Authority (Centro Nazionale Trapianti; CNT) issued specific guidelines and regulatory measures concerning donor management to all organ and tissue transplant facilities to prevent COVID-19 transmission. The evaluation of potential donors’ medical histories has been integrated with screening for SARS-CoV-2 by using quantitative real-time reverse-transcription PCR (qRT-PCR) on samples from nasopharyngeal swabs (NPS) or bronchoalveolar lavage (BAL) fluids, in accordance with the World Health Organization (WHO) recommendations [3].

Since solid organ and tissue transplantation are fundamentally necessary in order to save and improve patients’ lives, the criteria applied to ensure the suitability and safety of selected donors’ tissue need to be stricter in the current pandemic context [4]. Emerging data suggest that the sensitivity of a single negative test for SARS-CoV-2 may be insufficient to reliably exclude viral infection; often, subsequent tests (NPS, BAL) may provide positive results, especially when the specimen sample is obtained from a lower respiratory tract [5]. SARS-CoV-2 presence has been reported in several organ systems [6] and, while SARS-CoV-2 is mainly transmitted among humans via respiratory droplets, infection though organ and tissue transplantation cannot be excluded [7]. It is of note that, SARS-CoV-2 has been found in lung, spleen, heart, intestine, and liver tissues [8]; thus, the virus could be potentially present in all these donor organs [4]. This can be explained by the strong expression of the SARS-CoV-2 angiotensin-converting enzyme 2 (ACE2) receptor in different organs such as the heart (vascular endothelium of coronary arteries, vascular smooth muscle, and cardiomyocytes), kidneys (renal vessels and renal tubular epithelial cells), testes, and the gastrointestinal tract, which is likely to mediate SARS-CoV-2 entry [9].

Among possibly infected tissues, pulmonary heart valves and the contiguous pulmonary vein wall (PVW) play a central role in the cardiovascular field, as they are frequently transplanted tissues. In our Cardiovascular Tissue Bank (Milan, Italy), human pulmonary valve and PVW grafts are frequently required and commonly used for valve reconstruction. In comparison with bioengineered supports, the transplantation of human-donor-derived valves is still an advantageous surgery technique, especially in young patients with infective endocarditis and congenital heart diseases [10]. Indeed, valves and the contiguous PVW tissues possess ideal morphological and functional features such as growth potential during childhood, hemodynamic and rheological soundlessness, and longevity [11].

Data concerning the risk of SARS-CoV-2 transmission after tissue transplantation are, to date, controversial [12,13]. Recently, several SARS-CoV-2 inactivating procedures have been suggested in the literature for application during human tissue processing, but unfortunately, the most promising of these techniques (i.e., gamma irradiation) is not applicable to valve and PVW processing due to tissue injury [14]. Thus, in order to implement screening techniques, donor selection parameters, and to better evaluate the theoretical risk of virus transmission within valve transplantation, we tested SARS-CoV-2 presence in PVW samples, preserving pulmonary valve leaflet structures, as well as in other cardiovascular tissues collected at our tissue biobank in the period of April–May 2020 from NPS- and/or BAL-negative tissue donors considered eligible for pulmonary valve (PV) transplant following actual guidelines.

## 2. Materials and Methods

### 2.1. Tissue Procurement and Processing

Cardiovascular samples were collected during standard tissue bank operating procedures for transplantable pulmonary and aortic valve isolation.

Twenty-two human cardiovascular tissue specimens (8 from pulmonary vein wall, PVW; 2 from right atrium, RA; 2 from left atrium, LA; 2 from left ventricle, LV; 2 from interatrial septum, IAS; 2 from interventricular septum, IVS; 2 from aortic wall, AW; 2 from aortic valve leaflets, AVL), from 8 donors were included in the study. The serum samples of all 8 donors were kindly provided by ASST Papa Giovanni XXIII of Bergamo for serological assays.

The details of age, sex, cause of death, and specimen sampling time after death are listed in Table 1. The COVID-19-positive patient left ventricle samples were kindly provided by the Unit of Pathology of ASST Santi Paolo e Carlo of Milan.

### 2.2. Serological Test for the Detection of Antibodies Against SARS-CoV-2

Serum samples obtained from 7 out of 8 donors included in this study were run on the DiaSorin LIAISON XL Analyser instrument (Saluggia, Italy), using the DiaSorin LIASON SARS-CoV-2 S1/S2 IgG assay following manufacturer’s instructions. The assay is a chemiluminescent microparticle immunoassay (CMIA) for qualitative detection of IgG in human serum (or plasma) against the SARS-CoV-2 S1 and S2 proteins.

The sample of 1 out of the 8 donors included in this study was run on the Abbott Architect instrument (Chicago, IL, USA), using the Abbott SARS-CoV-2 IgG assay following manufacturer’s instructions. The test is a CMIA for qualitative detection of IgG antibodies against SARS-CoV-2 nucleocapsid protein in human serum and plasma. Strength of response in relative light units reflects quantity of IgG present and is compared to a calibrator to determine the calculated index (specimen/calibrator; [S/C]) for a sample (with positive at 1.4 or greater).

### 2.3. mRNA Extraction and qRT-PCR and Digital PCR Assays

RNA was extracted from tissues as previously described [15]. Briefly, every 100 mg of tissue sample was homogenized in 1 mL of TRIzol reagent using a TissueLyser (Qiagen, Hilden, Germany), following the manufacturer’s instructions. RNA pellets were resuspended in 32 μL RNAse-free water, quantified using a NanoDrop One (ThermoFisher Scientific, Waltham, MA, USA), and stored at −80 °C until further use. For qRT-PCR assay, retrotranscription was conducted on 400 ng of total RNA/sample using the 5X All-In-One RT MasterMix kit (ABM-Applied Biological Materials, Richmond, BC, Canada) following the manufacturer’s protocol. TaqMan probes for viral N gene and human RPLP0 gene (internal amplification control, IDT, Integrated DNA Technologies, Coralville, IA, USA) were used to detect viral RNA molecules using a ViiA 7 Real-Time PCR System. The amplification protocol was determined by applying the cycling conditions indicated for the commercial probes. The number of cycles of amplification was set to 60 in order to compensate for the possibility of reduced viral load in the samples. In order to increase detection accuracy, viral RNA quantification in tissue specimens was also performed on a QuantStudio 3D Digital PCR System platform using a one-step chip-based digital PCR. The platform was comprised the QuantStudio 3D Instrument, the Dual Flat Block GeneAmp PCR System 9700, and the QuantStudio 3D Digital PCR Chip Loader (ThermoFisher Scientific, Waltham, MA, USA). TaqMan Fast Virus 1-Step Master Mix (Applied Biosystems, Foster City, CA, USA) was used together with TaqMan primers and probes for viral N gene and human RPLP0 gene (internal amplification control, IDT, Integrated DNA Technologies, Coralville, IA, USA). Analyses were executed with the online version of QuantStudio 3D AnalysisSuite (Thermo Fisher Cloud, Waltham, MA, USA) following the manufacturer’s instructions.

### 2.4. Western Blot Analysis

Total tissue protein was extracted via tissue pulverizer and lysis buffer (Cell Signaling), supplemented with protease and phosphatase inhibitors, and quantified by BCA Protein Assay (ThermoFisher Scientific, Waltham, MA, USA). Primary antibodies adopted for Western blot analysis were specific for SARS-CoV-2 nucleoprotein (Cat#40143-R019, Sino Biological, Düsseldorf, Germany) and GAPDH (Santa Cruz Biotechnology, Dallas, TX, USA). GAPDH signal was used as the loading control.

## 3. Results

### 3.1. SARS-CoV-2 RNA Was Undetectable by qRT-PCR in Pulmonary Vein Wall Tissues of All Donors

All donor subjects involved in the study were negative for the molecular analyses of SARS-CoV-2 RNA presence in NPS and/or BAL, as reported in Table 1. In order to further evaluate the safety of organ donor tissues, we first analyzed the presence of antibodies against SARS-CoV-2 by serological assay on donor serum. Two out of eight donors (25%) tested positive for SARS-CoV-2 specific IgG, as shown in Table 1.

**Figure 1:**
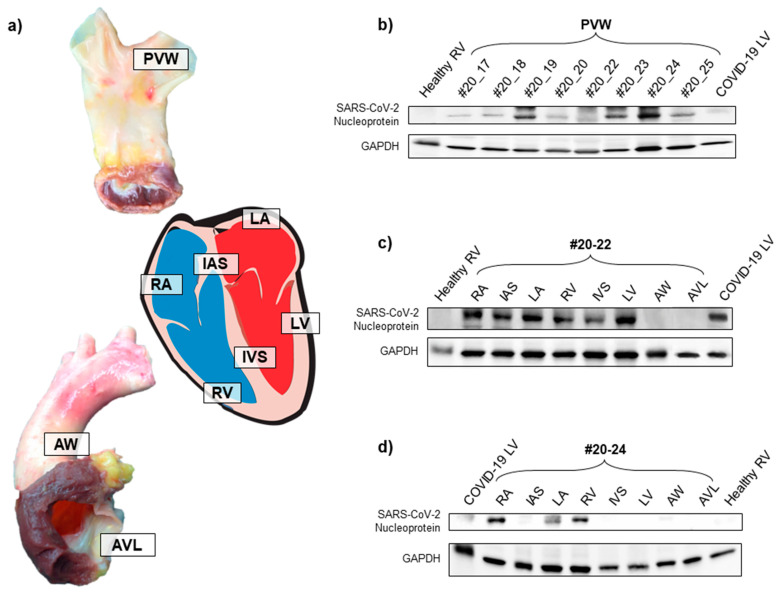
SARS-CoV-2 nucleoprotein was present in several tissues of COVID-19-negative donors. (**a**) Representative images of donor tissue specimens analyzed by Western blot. Labels in the images show the location of tissue sampling in each organ/tissue. (**b**) Western blot analysis for SARS-CoV-2 nucleoprotein on total protein extracts from tissues of 8 different deceased donor PVW specimens, RV specimens from healthy donors (collected prior to COVID-19 pandemic), and LV specimen from COVID-19-positive deceased patient. GAPDH was used as a loading control protein. (**c**,**d**) Western blot analysis for SARS-CoV-2 nucleoprotein on total protein extracts from different cardiac and valve tissues of 2 deceased donors (#20-22 and #20-24) positive for SARS-CoV-2 serological assay. RV specimen from a healthy donor (collected prior to COVID-19 pandemic) and LV specimen from a COVID-19-positive deceased patient were used as controls. GAPDH was used as the loading control protein. PVW: pulmonary vein wall; RA: right atrium; IAS: interatrial septum; LA: left atrium; RV: right ventricle; IVS: interventricular septum; LV: left ventricle; AW: aortic wall; AVL: aortic valve leaflet.

Subsequently, we checked for the presence of SARS-CoV-2 genome in RNA isolated from PWV samples of all donors (Figure 1a). Viral genome detection/quantification was then performed using two distinct techniques, qRT-PCR and chip-based digital PCR. None of the eight donors included in this study revealed SARS-CoV-2 presence, even in patient #20-22 and #20-24, who were both positive for the SARS-CoV-2 antibody serological test (Table 1).

These data suggest that, although two out of eight donors had surely been in contact with the virus, none of the subject samples showed SARS-CoV-2 RNA presence in PVW specimens.

### 3.2. SARS-CoV-2 Nucleoprotein Was Present in Pulmonary Vein Wall and Cardiac Tissues of Donors

In parallel, we examined the protein content of all PVW tissue in terms of SARS-CoV-2 nucleoprotein. In detail, we performed a Western blot analysis on total protein extract of PVW specimens from all donors (Figure 1b) and on several cardiac region samples from donors #20-22 and #20-24, who had returned a positive serological test (Figure 1c,d).

The analysis showed (i) the complete absence of SARS-CoV-2 nucleoprotein signal in healthy control samples and (ii) variable levels of nucleoprotein signal in all PVW donor samples, especially in donor #20-24. The in-depth analysis in cardiac specimens of donors #20-22 and #20-24 further highlighted the presence of SARS-CoV-2 nucleoprotein in other heart regions, especially in the right atrium and right ventricle (RA and RV). Importantly, in neither patient #20-22 nor patient #20-24 did we detect viral nucleoprotein signals in samples obtained from the thoracic aorta and aortic valve leaflets.

### 3.3. Late Results

Three out of the eight pulmonary valves collected were transplanted into recipient subjects between May and June of 2020. In September 2020, the recipients underwent an echocardiographic post-transplantation examination. None of the three recipient patients showed dysfunctions in the transplanted pulmonary valve. Importantly, none of the three recipients developed a symptomatic form of COVID-19. However, neither NPS nor serological analysis for SARS-CoV-2 antibody detection was performed.

## 4. Discussion

To the best of our knowledge, this is the first work reporting the presence of SARS-CoV-2 nucleoprotein in cardiovascular tissue recovered from tissue donors who died during the COVID-19 pandemic in Italy. Tissues analyzed in this study derived from subjects from the Italian region most exposed to SARS-CoV-2 virus (i.e., Lombardy) during the first pandemic peak. Our study fits in a framework in which debates on organ/tissue donation during COVID-19 pandemic period are still relevant due to the overall severity of this pandemic and to the appearance of a more severe second infection peak.

Interestingly, although all the enrolled subjects returned negative results to viral molecular tests on NPS and/or BAL, two out of eight returned positive results to the serological tests. Nonetheless, all subjects showed the presence of SARS-CoV-2 nucleoprotein in PVW protein extracts, even in the absence of viral RNA. A possible explanation for this situation is that the presence of the viral protein represents a kind of molecular “trace” left by SARS-CoV-2 past infection.

Surprisingly, we observed that some donors displayed the presence of viral protein not only in PVW, but also in several heart regions/tissues, from which a past infection of the entire cardiovascular region can be recognized. It is of note that the only fragments not showing viral nucleoprotein signal were the aortic valve leaflet and the aortic wall.

SARS-CoV-2 immunobiology (i.e., the development of specific antibodies) and the precise viral localization in different tissues are still not fully understood. The harshness of this COVID-19 pandemic is highlighted by the varied conditions of subjects involved; there were subjects who tested positive to serological assay after SARS-CoV-2 exposure that never developed symptoms (i.e., asymptomatic subjects) or asymptomatic subjects that negative to tests, who probably developed and displayed specific antibodies only during the infection period. Concerning the donors included in this study, it is important to highlight that they were asymptomatic subjects. Moreover, the viral transmission is affected by several epidemiological risk factors, incubation period, and viral load, as well as the viability of SARS-CoV-2 in blood and organs [16]. In this context, organ and tissue donors are a precious resource in the study of SARS-CoV-2 diffusion and the viral anatomical distribution.

Since the effects of SARS-CoV-2 on specific tissues are still not fully understood, and the potential impact of infected tissues on the recipient remains undefined, the criteria for donor selection in this time have been made stricter than before [4]. Recently, donor guidelines have been published in order to determine recommendations for all potential organ donors, such as SARS-CoV-2 RNA testing at collection and the discouragement of organ donation for all asymptomatic individuals who have been in a COVID-19-affected area for at least 28 days [17]. It is also important not to exclude the potential immunological response induced in recipients by SARS-CoV-2 nucleoprotein presence in the transplanted tissue, which may lead to anti-SARS-CoV-2 immunoglobulin production. Moreover, studies on autoptic samples of COVID-19 patients have clearly shown the presence of SARS-CoV-2 in the heart tissue [7,18,19,20,21]. In particular, our group reported the presence of SARS-CoV-2 RNA and nucleoprotein in the cardiomyocytes of COVID-19 patients without cardiac pathological involvement [7], suggesting an important role played by the post-discharge surveillance guidelines for surviving COVID-19 patients. Our present work confirms such literature by identifying viral proteins in the valve and heart tissue. However, the lack of viral RNA, as assessed by standard qPCR, used in diagnostics, and by the highly sensitive chip-based digital PCR, points to the safe use of the samples, as concerns guidelines.

The issue of safe tissue transplantation is not one with an easy solution, given the difficulties in implementing fast and tissue-specific diagnosis compatible with transplantation practice. Moreover, detailed organ-specific guidelines are needed, since the critical issues for transplant are dependent on both individual patient characteristics and the specific concerned organ [16].

## 5. Conclusions

In conclusion, our study demonstrated the presence of viral protein but not viral RNA in examined tissue bank samples and suggests several concerns. Firstly, it is important to thoroughly test via several molecular techniques not only organs, but also tissues, before transplantation. In particular, digital PCR and Western blot analyses on small specimens taken from close to the transplantable tissue may be indicated to help support biobank decisions. Moreover, this study also recommends caution in evaluating an entire organ as SARS-CoV-2 negative, since not all the tissue components may singularly result negative. Lastly, further studies will be necessary to dissect the potential long-term effects of SARS-CoV-2 nuclear protein in the receiving subjects.

## Figures and Tables

**Table 1:** Deceased donor details and results of SARS-CoV-2 antibody and RNA detection from serological tests and molecular assays.

Donor Code	Sex	Age	Cause of Death	Sampling Time (after Death)	N° Tests for Virus Detection	SARS-CoV-2 Antibody Detection	SARS-CoV-2 RNA in PVW (by qRT-PCR)	SARS-CoV-2 RNA in PVW (by Digital PCR)
#20-17	F	46	SH	4 h	1 NPS: NEG 3 BAL: NEG	3.99 AU/mL	undetectable	undetectable
#20-18	M	57	CA	17 h	1 NPS: NEG	<3.80 AU/mL	undetectable	undetectable
#20-19	F	64	SH	16 h	1 NPS: NEG 2 BAL: NEG	<3.80 AU/mL	undetectable	undetectable
#20-20	M	61	HT	7 h	1 NPS: NEG 1 BAL: NEG	<3.80 AU/mL	undetectable	undetectable
#20-22	F	61	IS	14 h	1 NPS: NEG 2 BAL: NEG	369 AU/mL	undetectable	undetectable
#20-23	M	50	CA	2 h	3 BAL: NEG	<3.80 AU/mL	undetectable	undetectable
#20-24	M	44	CA	14 h	1 NPS: NEG 2 BAL: NEG	8.21*	undetectable	undetectable
#20-25	M	54	CA	13 h	2 NPS: NEG 1 BAL: NEG	<3.80 AU/mL	undetectable	undetectable

Legend: F = female; M = male; SH = subarachnoid hemorrhage; CA = cardiac arrest; HT = head trauma; IS = ischemic stroke; NPS = nasopharyngeal swabs; BAL = bronchoalveolar lavage; PVW = pulmonary vein wall; * The relative light unit value is expressed as index [S/C].

## Data Availability

The raw data are available in Zenodo, at https://dx.doi.org/10.5281/zenodo.4452140.
